# Use of Atypical Antipsychotics as an Adjunct to the Treatment of Eating Disorders in Young People. Clinical Audit of Prescribing in the Children and Young People Eating Disorder Service Covering York, Scarborough, Harrogate and Northallerton, 2023

**DOI:** 10.1192/bjo.2024.644

**Published:** 2024-08-01

**Authors:** Natasha White

**Affiliations:** Tees, Esk and Wear Valleys NHS Foundation Trust, York, United Kingdom

## Abstract

**Aims:**

The prevalence of anorexia nervosa (AN) in young people is increasing and it is the psychiatric condition with the highest morbidity and mortality. Atypical antipsychotics are unlicensed for use for AN but research has demonstrated they can improve weight restoration and decrease anxiety and rumination around food and body image. However, prescription of antipsychotics comes with risks such as arrythmias, hyperprolactinaemia or EPSEs.

As a result, NICE (National Institute of Clinical Excellence) and TEWV (Tees, Esk and Wear Valleys) Trust have developed standards to ensure the safety and efficacy of antipsychotic prescribing in young people with anorexia nervosa. This audit aims to measure local compliance with these standards by reviewing antipsychotic prescribing and monitoring in the Children and Young People (CAMHS) Eating Disorder Service covering North Yorkshire.

**Methods:**

Clinicians identified all patients under the team currently prescribed an antipsychotic for AN; 8 patients in total. Electronic patient records were hand searched for relevant information.

Standards were derived from RCPsych May 2022: Medical Emergencies In Eating Disorders and local TEWV Guidelines.

Initial data collection was June 2023. Recommendations included a spreadsheet to monitor antipsychotic prescription and advice on documentation of unlicensed indications and provision of medication information. Re-audit was December 2023. Patients included in the initial audit were excluded to avoid duplication of results; 3 patients were in the re-audit.

**Results:**

All patients received full eating disorder assessment and non-pharmacological interventions such as dietetic or psychological input. All patients were prescribed olanzapine. The indication of antipsychotic prescription for all patients was distress/rumination. Compliance with physical health monitoring was 100% in both audits. Compliance with blood-test monitoring was initially 17%, rising to 100% in re-audit. 50% of patients were given medication information, increasing to 67% in re-audit. Compliance with local protocol for unlicensed indications was 13% and 0% in re-audit. Psychiatric follow up and medication review was regular and consistent in both initial and re-audits.

**Conclusion:**

Physical health monitoring was very good, likely due to routine AN monitoring regardless of antipsychotic prescription. Psychiatric review was frequent, reflecting the intensity of CAMHS practice. Initial poor compliance with blood-test monitoring was due to antipsychotic-specific blood tests being omitted from standard tests. Overall compliance improved after implementation of recommendations, particularly blood-test monitoring. Ongoing areas for improvement are following the protocol for unlicensed medication indications and provision of additional medication information. Most patients had psychiatric comorbidities and co-prescribed psychotropic medication, reflecting the complexity and severity of this cohort.

